# Redox imbalance and depression: the emerging role of molecular hydrogen in neuroprotection

**DOI:** 10.4103/mgr.MEDGASRES-D-25-00105

**Published:** 2026-09-12

**Authors:** Francisco Javier Liévanos-Ruiz, Bertha Fenton-Navarro

**Affiliations:** Laboratorio de Glicobiología y Farmacognosia, División de estudios de Posgrado, Facultad de Ciencias Médicas y Biológicas “Dr. Ignacio Chávez”, Universidad Michoacana de San Nicolás de Hidalgo (UMSNH), Morelia, Michoacán, México; Laboratorio de Glicobiología y Farmacognosia, División de estudios de Posgrado, Facultad de Ciencias Médicas y Biológicas “Dr. Ignacio Chávez”, Universidad Michoacana de San Nicolás de Hidalgo, Av. Dr. Rafael Carrillo S/N, Esq. Dr. Salvador González Herrejón, Bosque, Cuauhtémoc, Morelia, Michoacán, Mexico

##  

Major depressive disorder is a multifaceted psychiatric condition with profound personal, social, and economic consequences. It affects hundreds of millions of people worldwide and stands as a leading cause of disability.^1^,^2^,^3^ Although antidepressant medications have improved the clinical management of this disorder, a significant proportion of patients do not respond adequately to first-line pharmacotherapy.^2^,^3^ This subset, often referred to as treatment-resistant depression, highlights the pressing need for novel therapeutic approaches that move beyond the traditional focus on monoaminergic systems.^2^,^3^

In recent years, biological processes such as oxidative stress, neuroinflammation, and mitochondrial dysfunction have gained prominence as critical elements in the pathophysiology of depression.^1^,^2^,^3^ Among these, oxidative stress has drawn particular interest due to its role in neuronal damage, impaired neuroplasticity, and the disruption of neurotransmitter signaling.^1^,^2^,^3^ Despite its importance, oxidative stress remains insufficiently addressed by current treatments.^2^

One emerging candidate capable of modulating these biological mechanisms is molecular hydrogen.^4^,^5^ Once thought to be biologically inert, hydrogen has demonstrated selective antioxidant and anti-inflammatory properties in diverse experimental models.^4^,^5^ Its small molecular size enables it to permeate cellular compartments and cross the blood-brain barrier, offering potential therapeutic benefits in neuropsychiatric conditions.^4^,^5^ This article presents a scientific perspective on the role of molecular hydrogen as a possible treatment for depression. It argues that hydrogen, by targeting key pathological pathways, may offer a safe and biologically plausible approach for future clinical interventions.

**The role of oxidative stress in depression:** The central nervous system is particularly susceptible to oxidative damage. This vulnerability is due to its high oxygen demand, abundant lipid content, and relatively limited antioxidant defenses. In major depressive disorder, numerous studies have documented increased levels of oxidative biomarkers, including lipid peroxidation products, oxidized DNA bases, and protein carbonyls.^1^,^2^,^3^ These alterations have been observed in both peripheral tissues^1^,^3^ and postmortem brain samples.^2^,^3^ Alterations in the levels of endogenous antioxidant enzymes such as superoxide dismutase, glutathione peroxidase, and catalase have been reported in major depressive disorder, although findings have been inconsistent across studies. Some reports describe reduced activity, while others suggest compensatory increases, particularly during acute episodes.^1^,^2^,^3^

Oxidative stress is not a mere consequence of depression. It plays an active role in the initiation and perpetuation of the disorder.^1^,^2^,^3^ Reactive oxygen species can damage mitochondrial DNA, impair electron transport, and reduce adenosine triphosphate production.^1^,^3^ This compromises neuronal viability and synaptic transmission, particularly in brain regions involved in mood regulation, such as the hippocampus and prefrontal cortex.^6^

Oxidative stress has been shown to impair key neuroplastic processes involved in the pathophysiology of depression.^1^,^2^,^3^ It contributes to disruptions in synaptic function and plasticity, in part by reducing levels of neurotrophic factors such as brain-derived neurotrophic factor (BDNF).^2^,^3^,^7^ Lower BDNF expression has been associated with impaired dendritic remodeling and reduced adaptive responses to stress. These alterations may underlie deficits in cognitive flexibility and emotional regulation commonly observed in depression.^7^ Furthermore, elevated oxidative stress has been linked to poorer treatment outcomes, particularly in patients with treatment-resistant depression.^2^

Notably, oxidative stress is closely interconnected with neuroinflammation.^1^,^2^,^3^ Reactive oxygen species activate redox-sensitive transcription factors, including nuclear factor kappa B. This leads to the production of pro-inflammatory cytokines that exacerbate oxidative damage and further compromise neuronal integrity.^2^,^3^ Inflammatory cytokines, in turn, alter monoamine metabolism, reduce serotonin availability, and disrupt neurogenesis.^3^ This bidirectional relationship creates a pathological feedback loop that reinforces depressive symptoms and reduces treatment responsiveness.^2^

**Molecular hydrogen as a selective antioxidant and anti-inflammatory agent:** Hydrogen is the smallest and most abundant molecule in the universe (**Figure 1**). In biological systems, it has the unique ability to act as a selective antioxidant.^4^,^5^,^8^ Unlike conventional antioxidants that may interfere with essential redox signaling, molecular hydrogen specifically targets the most harmful reactive species, such as radicals and peroxynitrite, while leaving less reactive molecules unaltered.^4^,^5^,^8^

**Figure 1:**
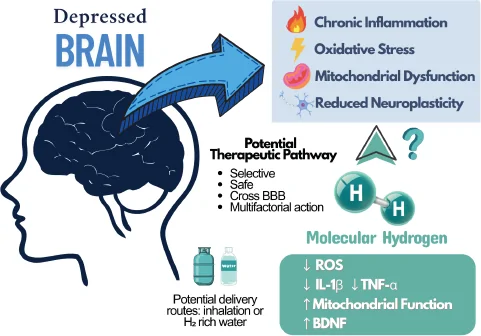
Potential neuroprotective role of molecular hydrogen in depression. BBB: Blood-brain barrier; BDNF: brain-derived neurotrophic factor; IL: interleukin; ROS: reative oxygen species; TNF: tumor necrosis factor.

This selective action allows hydrogen to mitigate oxidative stress without disrupting physiological processes.^4^,^5^,^8^ Additionally, hydrogen activates cellular defense systems by stimulating the nuclear factor erythroid 2-related factor 2 pathway.^5^,^8^ This leads to the transcription of cytoprotective enzymes involved in redox homeostasis. These include heme oxygenase 1, superoxide dismutase, and glutathione-related enzymes.^5^

Hydrogen also exhibits significant anti-inflammatory properties.^4^,^5^,^8^ It inhibits the production of tumor necrosis factor alpha, interleukin 1 beta, and interleukin 6.^5^,^8^ These cytokines are frequently elevated in individuals with depression and are known to interfere with neurotransmission and plasticity.^7^ By reducing cytokine levels.^5^,^8^ Hydrogen may help restore immune balance and prevent inflammation-induced neurotoxicity.

Preclinical studies have demonstrated the protective effects of hydrogen in various neurological conditions, including cerebral ischemia,^4^,^5^,^8^ traumatic brain injury, Parkinson’s disease, and Alzheimer’s disease.^4^,^5^,^8^ In these models, hydrogen administration has led to reduced oxidative damage, improved mitochondrial function, and preservation of cognitive and behavioral functions.^5^,^8^ Although the evidence in depression-specific models is limited, the biological pathways targeted by hydrogen strongly overlap with those implicated in the pathogenesis of major depressive disorder.^5^,^8^

**Neurobiological relevance to depression:** Hydrogen’s potential relevance to depression lies in its ability to address oxidative imbalance, inflammation, and mitochondrial dysfunction simultaneously.^5^,^8^ These factors are central to the neurobiology of depression and are particularly relevant in treatment-resistant cases.^2^

One of the key mechanisms by which hydrogen may exert antidepressant effects is through the preservation of BDNF.^5^,^8^ Oxidative stress and inflammation are known to downregulate BDNF expression. Reduced BDNF levels have been associated with impaired synaptic plasticity, reduced hippocampal volume, and poor response to antidepressants.^3^ By attenuating oxidative and inflammatory insults, hydrogen could help to maintain or restore BDNF signaling.^5^,^8^

Another important mechanism involves the modulation of the kynurenine pathway. Inflammatory processes in depression enhance the activity of indoleamine 2,3-dioxygenase, an enzyme that diverts tryptophan metabolism away from serotonin synthesis and toward the production of neurotoxic metabolites such as quinolinic acid. These metabolites can overstimulate glutamate receptors and promote excitotoxicity.^9^ Hydrogen, by reducing pro-inflammatory cytokines,^5^,^8^ may help normalize tryptophan metabolism and prevent downstream neurotoxicity.

Hydrogen also has beneficial effects on mitochondrial function,^5^,^8^ which is often compromised in depression. Mitochondria are central to neuronal energy metabolism, calcium signaling, and the regulation of apoptotic pathways.^5^,^8^ Oxidative damage to mitochondrial membranes and DNA impairs their function, leading to reduced adenosine triphosphate production and increased vulnerability to cell death.^5^,^8^ Experimental data suggest that hydrogen can preserve mitochondrial integrity, reduce reactive oxygen species production, and enhance bioenergetic efficiency.^5^,^8^

Beyond these molecular targets, the cellular environment also plays a critical role in neurobiological impact of the hydrogen. Microglia play a central role in neuroinflammation, shifting toward a proinflammatory M1 phenotype that promotes cytokine release and oxidative damage. Molecular hydrogen has been shown to suppress M1 polarization and reduce nuclear factor kappa B-mediated responses while activating the Nrf2 antioxidant pathway. In astrocytes, hydrogen attenuates reactive astrogliosis and supports neuroprotective A2 phenotypes. Neurons, particularly vulnerable to oxidative injury, benefit from hydrogen’s capacity to reduce reative oxygen species, preserve mitochondrial integrity, and prevent apoptosis. These cellular effects may underlie hydrogen’s potential to modulate mood-related circuits and improve stress resilience.^5^,^8^

In **Figure 2**, the neuroprotective mechanisms of molecular hydrogen in major depressive disorder are summarized.

**Figure 2:**
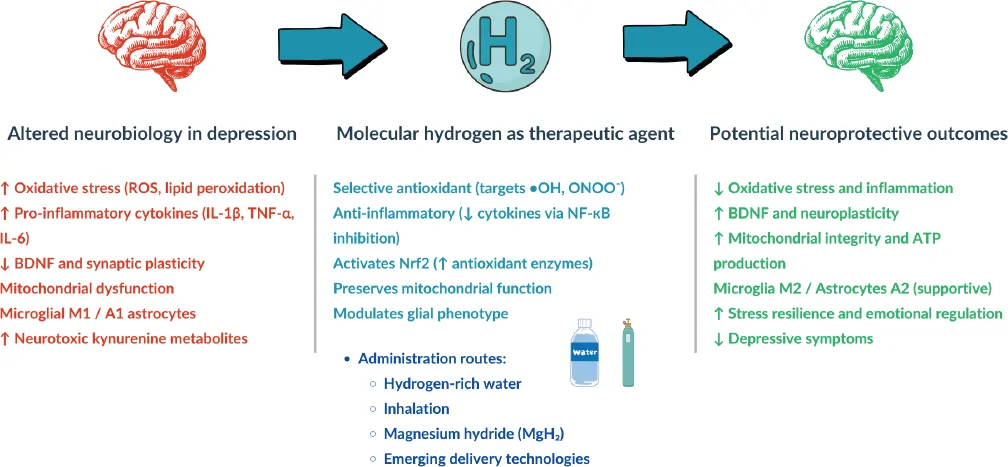
Neuroprotective mechanisms of molecular hydrogen in major depressive disorder. ATP: Adenosine triphosphate; BDNF: brain-derived neurotrophic factor; IL: interleukin; NF-κB: nuclear factor kappa B; Nrf2: nuclear factor erythroid 2-related factor 2; ROS: reative oxygen species; TNF: tumor necrosis factor.

**Translational considerations and research directions:** The transition from preclinical promise to clinical application requires careful consideration.^4^,^5^,^8^ While the safety profile of hydrogen is well established,^4^,^8^ key questions remain regarding its therapeutic potential in psychiatric populations.^5^ To address these, a multiphase research strategy is warranted.

First, further preclinical studies in animal models of depression should assess behavioral, neurochemical, and molecular outcomes following hydrogen administration. These studies should compare different delivery methods, including inhalation and hydrogen-rich water and evaluate dose-response relationships.^4^ Biomarkers of oxidative stress, inflammation, and neuroplasticity should be included to elucidate mechanisms of action.

Second, early-phase clinical trials in humans are essential to evaluate safety, tolerability, and preliminary efficacy. These trials should recruit patients with well-characterized depression, including treatment-resistant cases, and incorporate biological assessments to identify responders. Ideally, such studies would compare hydrogen therapy as both a monotherapy and an adjunct to standard antidepressants. In addition to conventional administration methods, novel hydrogen-releasing materials such as magnesium hydride (MgH_2_) offer new avenues for clinical application.^4^,^10^ MgH_2_ provides controlled and sustained hydrogen release and has shown antidepressant-like effects in animal models by reducing neuroinflammation, oxidative stress, and microglial activation.^10^ From a translational perspective, such materials could improve hydrogen bioavailability and therapeutic consistency. Ongoing advances in delivery technologies may facilitate the development of more practical and targeted interventions for mood disorders.

Third, the long-term effects of hydrogen treatment remain largely unknown. Future research should investigate whether continuous or intermittent administration is more effective, and whether there are cumulative benefits or risks over time. Longitudinal designs and follow-up assessments will be crucial for answering these questions.

Finally, advances in personalized medicine may help tailor hydrogen therapy to specific subgroups. For example, patients with elevated oxidative biomarkers or inflammation-related genetic profiles may derive greater benefit from antioxidant interventions. Identifying these individuals in advance could improve treatment outcomes and reduce unnecessary exposure.

**Conclusion:** Molecular hydrogen represents a promising therapeutic candidate for the treatment of major depressive disorder. Its unique ability to selectively neutralize toxic reactive species, reduce inflammation, and protect mitochondrial function aligns with key pathophysiological processes in depression. While current evidence is preliminary, the strong mechanistic rationale and favorable safety profile justify further investigation.

Future studies should focus on refining delivery methods, identifying responsive subpopulations, and establishing efficacy in clinical trials. In a field where many patients remain underserved by existing therapies, hydrogen offers a novel and potentially transformative approach to restoring brain health and emotional well-being.

*This work was partially supported by Secretaría de Ciencia, Humanidades, Tecnología e Innovación (SECIHTI); postdoctoral fellowship CVU: 590007 and CIC-UMSNH-BFN-2025*.

*The authors declare that no generative AI was used in the preparation of this manuscript.*
